# Predictors of Informal Caregiver Burden in Parkinson’s Disease: A Systematic Review

**DOI:** 10.1177/01939459251327968

**Published:** 2025-03-26

**Authors:** Rosie Lesley, Jane Simpson, Maria Dale, Fiona Eccles, Selina Lock, Sarah Gunn

**Affiliations:** 1 University of Leicester, Leicester, UK; 2 Lancaster University, Lancaster, UK; 3 Leicestershire Partnership NHS Trust, Leicester, UK

**Keywords:** Parkinson disease, caregivers, caregiver burden, systematic review

## Abstract

**Background:**

Caregivers of people with Parkinson’s disease are at risk of experiencing *caregiver burden*. Understanding contributing factors is required to develop appropriate targeted interventions and support for this group. This systematic review provides an updated appraisal and synthesis of quantitative studies assessing predictors of burden among informal caregivers of people with Parkinson’s.

**Method:**

Five electronic databases (APA PsycINFO, CINAHL, MEDLINE, Web of Science, and Cochrane Library) were systematically searched (from inception until July 2024), supplemented by hand-searches. Study quality was assessed using the cross-sectional JBI Critical Appraisal Checklist. Results were synthesized narratively.

**Results:**

Forty-one studies were included. Predictors of increased burden included greater impact of motor symptoms on activities of daily living, greater severity of neuropsychiatric symptoms, poorer quality of life of the person with Parkinson’s, and poorer caregiver mental health. Demographics, presence of motor symptoms, motor complications, and general cognitive function did not predict burden. Evidence was inconclusive for several variables including disease stage and duration, motor symptom severity, functional ability, overall non-motor symptoms, mental health of the person with Parkinson’s, and caregivers’ involvement and protective factors.

**Conclusion:**

Several areas for potential future intervention are indicated, although methodological weaknesses within the literature constrain the robustness of conclusions. Key areas for future research include exploring understudied variables (caregiver personality and coping style, relationship quality, and positive aspects of caregiving) that may be important predictors of burden, specifying and utilizing a more consistent definition of “informal caregiver,” and recruiting younger and non-spousal caregivers and more diverse samples regarding disease severity.

Parkinson’s disease is the second most common neurodegenerative condition after Alzheimer’s disease,^1^ currently affecting an estimated 9.4 million people worldwide.^2^ It is characterized by motor symptoms including bradykinesia, rigidity, rest tremor, and postural instability,^3^ which can be used to differentiate Parkinson’s from other parkinsonian disorders.^4^ Other features of Parkinson’s disease can include sleep problems, psychological difficulties (eg, depression, anxiety, apathy, and psychosis), impulsivity, cognitive impairment and dementia, and autonomic dysfunction.^5^

As Parkinson’s progresses, individuals typically require increasing support and assistance with daily tasks,^6^ including medication management, activities of daily living (ADL), personal safety, care coordination, and social activities.^7,8^ Since most people with Parkinson’s (PwP) live in the community,^9^ informal caregivers (people who are not financially compensated for providing care, typically spouses/partners or other family members) are usually main providers of their care.^10^ This can become challenging as the disease progresses, especially given a lack of services^11^ and/or perceived lack of coordinated support from health care services.^12^

Caring for a PwP can affect various aspects of a caregiver’s life.^12,13^ This is often termed *caregiver burden* (CB),^9^ although other terms are also used to describe similar phenomena, such as *stress*,^14^ *distress*,^15^ or *strain*.^16^ The concept of CB has been operationalized in various ways (for reviews, see Chou^17^ and Liu et al^18^), most often as a broad, multidimensional construct describing “the extent to which caregivers perceived their emotional or physical health, social life, and financial status as suffering as a result of caring for their relative.”^19(p261)^

Regarding terminology, although commonly used, “caregiver” and “caregiver burden” are contentious terms, argued to be culturally-biased,^20,21^ negatively-valenced, disregarding the caring and reciprocal component of relationships,^22-25^ and bureaucratizing of a common human experience.^26,27^ Similarly, “caregiver burden” risks engendering a blaming or dismissive perspective toward the “cared-for,”^28-30^ potentially (unintentionally) prioritizing caregivers’ needs.^31^ While the term “carer” made some headway in promoting the recognition of care work,^32^ it carries inherent biases,^21^ and using relationship-focused labels is generally preferred by the described populations.^24^ We use “caregiver” and “caregiver burden” cautiously in this review given their ongoing ubiquity, in the absence of a commonly accepted alternative.

## Caregivers in the Context of Parkinson’s

Although caregivers of people with other chronic conditions experience some similarities in burden to those supporting a PwP,^9^ the latter bring unique challenges due to its progressive nature, myriad of motor symptoms and other difficulties, and frequently fluctuating symptoms.^33,34^ Parkinson’s motor symptoms usually develop between 65 and 70 years of age,^35^ coinciding with the typical time of transitioning to retirement, and causing disruption and loss of long-held future plans for the affected person and family.^8^ Furthermore, as the disease progresses, informal caregivers may experience changed roles within relationships, anxiety about the future, grief, frustration, guilt, reduced involvement in work and social activities, financial pressures (predominantly through the loss of income), and struggle with access to services and/or poorly coordinated health care.^8,11,12,36,37^ These circumstances can negatively impact caregivers’ physical, psychological, and social well-being^13,38-43^ and reduce their quality of life (QoL).^44,45^

Ensuring caregiver well-being is paramount in supporting PwP. High CB can lead to burnout,^46^ producing an “invisible patient” in the patient-clinician-caregiver system and reducing a caregiver’s ability to continue providing effective care.^8^ This, in turn, adversely affects health outcomes of PwP.^47^ Furthermore, although many PwP wish to live at home for as long as possible, CB is a risk factor for the PwP entering a care home.^36,48,49^ Informal caregivers also contribute substantially to society; the economic value of their care and support is greater than ever, and without them, health and social care systems would collapse.^50^ Recently, the COVID-19 pandemic has exacerbated rates of CB in Parkinson’s through the loss of professional and family support and social isolation.^51,52^ Accordingly, understanding factors that contribute to burden among informal caregivers of PwP is crucial to developing targeted supportive interventions focused on preventing or reducing their experienced burden and supporting them to maintain well-being and effectiveness in their role.

### Previous Reviews

Numerous studies have investigated factors associated with CB in Parkinson’s. Six quantitative reviews have provided overviews of this literature.^8,15,53-56^ In 2015, Greenwell et al^54^ synthesized and evaluated evidence on predictors of psychosocial outcomes (including burden) in Parkinson’s caregivers. Since 2015, a substantial number of relevant articles have been published, so a fresh review is timely. Other reviews in the area have either focused on a specific method (longitudinal studies and only searching in PubMed)^55^ or a specific short time period (2017-2022).^53^ Consequently, an updated comprehensive synthesis of literature regarding factors related to CB for those supporting a PwP is necessary.

### Review Aims

Accordingly, this systematic review provides an updated appraisal and synthesis of evidence around factors associated with CB among informal caregivers of PwP. The focus is on predictors (rather than correlates) of informal CB, examining the direction of the relationship between variables and identifying implications of clinical relevance for potential supportive interventions.

## Methods

### Search Strategy

The electronic databases APA PsycINFO, CINAHL Plus with Full Text, MEDLINE, Web of Science Core Collection, and Cochrane Library were systematically searched on July 25, 2023, using a combination of subject headings and search terms relating to “Parkinson’s disease,” “burden,” and “caregiver” (Table 1), which were combined using the Boolean operators “OR” and “AND.” The search strategy was developed based on a prior scoping search and in consultation with a specialist university librarian. Searches were limited to peer-reviewed articles, written in English, and where possible a restriction was added to only retrieve papers with human participants. No limits were placed on year of publication. Additionally, reference lists of relevant review articles returned from the initial search and articles meeting the inclusion eligibility criteria were manually searched. An updated search was conducted on July 23, 2024, limited to articles published since January 1, 2023.

**Table 1.** Terms for Systematic Literature Search.

Subject headings	Title, abstract, and keyword searches
Parkinson’s disease	parkinson*
Caregiver burden	burden or stress or distress or strain
Caregivers	carer* or caregiv* or “care giv*” or family or families or relatives or spous* or husband* or wife or wives or partner or partners

### Inclusion and Exclusion Criteria

Inclusion and exclusion criteria for studies are summarized in Table 2. Findings were only included from multivariable analyses (ie, statistical models that have multiple independent or predictor variables and a single dependent or outcome variable).^57^ Bivariate statistical analyses are argued to be inadequate for investigating complex constructs such as CB,^58^ with multivariable statistics offering a better understanding of the unique contributions of many variables that may influence burden.^59,60^

**Table 2.** Inclusion and Exclusion Criteria for Studies.

Inclusion criteria	Exclusion criteria
1) The study’s sample consisted only of informal (unpaid) caregivers of people with Parkinson’s (at any disease stage), with any relationship to the person with Parkinson’s (eg, spouse/partner, sibling, friend), who had been caring for any duration.	1) The study’s sample included formal (paid) caregivers of people with Parkinson’s, or caregivers of other populations.
2) Main aim of the study was to identify caregiver or person with Parkinson’s factors predicting caregiver burden among informal caregivers of people with Parkinson’s through multivariable statistical modeling.	2) Mixed-sample studies (eg, informal caregivers of people with different conditions grouped together, unless subsample analysis enabled discrimination of data regarding those supporting a person with Parkinson’s).
3) The study reported a measure of burden among informal caregivers of people with Parkinson’s using a total score on a validated caregiver burden scale.	3) The study only investigated correlations.
4) Caregiver and person with Parkinson’s factors explicitly defined; self-reported constructs measured with a validated questionnaire or clearly described question.	4) The study included predictors or outcomes measuring multiple constructs (eg, using factor analysis; combining depression and anxiety into one overall predictor; combining burden with other constructs into one outcome measure).
5) Quantitative methodology, or mixed-methods with sufficient quantitative component for the extraction of quantitative results.	5) Caregiver burden was an independent variable instead of the outcome.
6) Original empirical study in a peer-reviewed journal, full-text article available, in English (funds unavailable for translation).	6) Intervention study, qualitative study, case study, review, editorial, or scale validation.

### Study Selection

**Figure 1.**
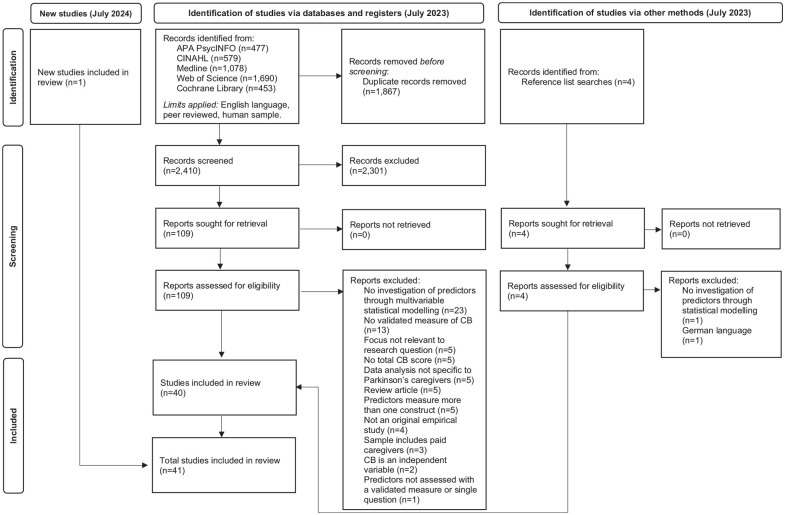
PRISMA flow diagram of the systematic search procedure.

The initial search identified 4277 records after limits were applied. Articles were imported into the EndNote reference management software (Clarivate, Philadelphia, PA, USA), and 1867 duplicates were removed. Titles and abstracts of the remaining 2410 articles were screened for relevance. After this, 109 articles were retrieved for full-text screening against the eligibility criteria, and 38 selected for inclusion. Two further articles were identified through hand-searching reference lists of relevant review articles returned from the initial search. The online platform Rayyan^61^ was used to support the screening process and record decision-making. Figure 1 depicts the systematic search PRISMA diagram.^62^

### Data Extraction and Quality Appraisal

A data extraction tool was developed to extract information relevant to the review. Extracted data included author, publication year, country of study, aim, main definitions, participant characteristics, recruitment methods, study design, outcome measures, statistical analysis, and key findings.

Since all included studies utilized a cross-sectional design, methodological quality appraisal was undertaken using the JBI Critical Appraisal Checklist for Analytical Cross-Sectional Studies.^63^ The tool requires reviewers to indicate whether a study meets 8 criteria by selecting *Yes*, *No*, *Unclear*, or *Not applicable* for each of 8 questions. The first author independently assessed quality of selected studies using the checklist, with ratings for 10% then cross-checked by another author to ensure reliable quality appraisal. Any differences of opinion were resolved via discussion. The checklist does not yield a score to indicate methodological quality; indeed, it has been argued a pure numerical outcome does not best reflect methodological quality and can mask significant issues in specific areas.^64-66^ Following JBI guidelines,^63^ the tool was not used to exclude articles based on low methodological quality. Instead, appraisal results were used to highlight quality of the current evidence base and inform interpretation of findings.

### Data Synthesis

Due to heterogeneity of the studies with a wide range of outcome measures used to assess potential predictors and CB, it was not appropriate to conduct a meta-analysis. Instead, a narrative approach^67^ was used to synthesize evidence regarding caregiver and PwP-related predictors of burden in informal caregivers of PwP.

## Results

### Characteristics of Included Studies

Forty-one full-text articles were identified for inclusion (key features summarized in Table 3). They were published between 1997 and 2023, the majority (35 articles) since 2012. Studies were conducted in Germany (n = 5), India (n = 5), the United Kingdom (n = 5), Italy (n = 3), South Korea (n = 3), the United States (n = 3), Australia (n = 2), Brazil (n = 2), Spain (n = 2), Sweden (n = 2), Malaysia (n = 1), Mexico (n = 1), the Netherlands (n = 1), Poland (n = 1), Thailand (n = 1), and Turkey (n = 1). Three multinational studies were conducted across the United States and Canada,^68^ the United States and Japan,^69^ and the United Kingdom, France, Germany, the Netherlands, Portugal, and Sweden.^70^ All studies used a cross-sectional design. Thirty-two were designed as single studies and 9 were substudies that analyzed baseline data.^47,68,70-76^ Two studies shared a database; however, both were included as they contained separate statistical analyses.^77,78^

**Table 3.** Study Characteristics and Key Findings.

Authors (year); country	Study characteristics			Caregiver characteristics			Identified predictors of caregiver burden
	Caregiver sample size	Recruitment^a^	Statistical analysis	Spouse/partner of PwP (%)	Female (%)	Mean age (years)	
Agrawal et al (2012)^79^; India	91	Medical setting	Multiple regression	49.5	54.9	44.6	Caregiver: Number of caregivers: β = −0.311, *P* = .000 PwP: Sleep disturbance: β = 0.206, *P* = .025 UPDRS III: β = 0.255, *P* = .004 BDI: β = 0.352, *P* = .000
Bartolomei et al. (2018)^80^; Italy	55	Medical setting	Multiple regression	85.5	NR	62	PwP: PDQ-39: *R*^2^ = 0.38, *P* < .001
Caap-Ahlgren and Dehlin (2002)^73^; Sweden	65	Medical setting	Multiple regression	89.2	36.9	67	Caregiver: GDS: *B* = 1.30, *P* = .014 SOC: *B* = −0.35, *P* = .004 PwP: HY: *B* = 4.67, *P* = .001
Carod-Artal et al. (2013)^81^; Brazil	50	Medical setting	Hierarchical multiple regression	78	88	55.7	Caregiver: Caregiving duration: β = 0.38, *P* = .002 HADS-Anxiety: β = 0.34, *P* = .006 PwP: SCOPA-Sleep: β = 0.38, *P* = .01 PPRS: β = 0.29, *P* = .008
Carrilho et al. (2018)^82^; Brazil	21	Medical setting	Multiple regression	47	80	53	No significant predictors
D’Amelio et al. (2009)^83^; Italy	40	Medical setting	Stepwise multiple regression	100	NR	63.6	PwP: HY: β = 0.62, *P* < .001 NPI-12: β = 0.28, *P* = .03
Edwards and Scheetz (2002)^84^; United States	41	Medical setting; community	Stepwise multiple regression	100	68	66.8	Caregiver: PSS-Fa: β = −0.317, *P* = .037 PwP: ADL-MS: β = −0.435, *P* = .005
Eichel et al. (2022)^85^; Germany	84	Medical setting	Multiple regression	94	64.3	66.2	PwP: SEND-PD (Mood/Apathy): β = 0.444, *P* = .001
Geerlings et al. (2023)^47^; the Netherlands	504	Medical setting	Multiple regression	90.7	66.9	67.6	Caregiver: MSPSS: β = −0.108, *P* < .001 COPE-28 (Problem-focused): β = 0.352, *P* < .001 COPE-28 (Avoidant): β = 0.973, *P* < .001 PwP: Work status (working): β = −0.254, *P* < .05 MDS-UPDRS II: β = 0.251, *P* < .05 SPDDS: β = −0.011, *P* < .05
Goel et al. (2022)^86^; India	145	Medical setting	Multiple regression	54.8	NR	46.2	Caregiver: Number of caregivers: β = −0.506, *P* < .001 PwP: Age: β = 0.388, *P* < .001 Disease duration: β = 0.434, *P* < .001 HY: β = 0.711, *P* < .001 PDQ-39: β = 0.676, *P* < .001 NPI-12: β = 0.883, *P* < .001 NPI-12 Domains—Delusion: β = 0.533, *P* < .001 Hallucinations: β = 0.690, *P* < .001 Agitation: β = 0.493, *P* < .001 Depression: β = 0.209, *P* = .046 Anxiety: β = 0.332, *P* = .004 Elation: β = 0.255, *P* = .029 Disinhibition: β = 0.337, *P* = .004 Irritability: β = 0.318, *P* = .006 Aberrant motor behavior: β = 0.288, *P* = .015 Sleep disturbances: β = 0.410, *P* < .001 Appetite and eating disorders: β = 0.394, *P* < .001
Golińska et al. (2017)^87^; Poland	20	Community	Hierarchical multiple regression	NR	80	61.9	Caregiver: SOC-Manageability: β = −0.52, *P* = .01 PwP: Disease duration: β = 0.38, *P* = .02 Verbal fluency (phonemic): β = −0.68, *P* = .01
Hand et al. (2022)^88^; UK	115	Medical setting	Stepwise multiple regression	85.2	66.1	70.7	Caregiver: Health: *B* = 7.165, *P* < .001 SCOPA-carer nighttime sleep: *B* = 0.532, *P* = .022 COPE-28 (Active): *B* = 1.755, *P* = .004 RAS: *B* = −0.726, *P* < .001 PwP: NPI-12 (Motor Behavior): *B* = 6.997, *P* = .036 UPDRS-eating: *B* = 3.447, *P* = .002 PDQ-39 (Mobility): *B* = 0.073, *P* = .036 Bathing: *B* = 6.676, *P* < .001 Mealtimes: *B* = 5.948, *P* = .002
Johnson et al. (2023)^71^; UK	45	Medical setting	Backward stepwise multiple regression	55.6	NR	NR	Caregiver: GHQ-28: β = 0.425, *P* = .003 PwP: NPI-12: β = 0.319, *P* = .023
Jose et al. (2021)^89^; India	104	Medical setting	Multiple regression	51	76.9	NR	Caregiver: Caregiving duration: β = 0.276, *P* = .001 PwP: EASI: β = 0.158, *P* = .038 NPI-12: β = 0.509, *P* = .001
Kalampokini et al. (2022)^70^; UK, France, Germany, Netherlands, Portugal, Sweden	506	Medical setting	Stepwise multiple regression	71.2	NR	NR	PwP: NPI-12: β = 0.331, *P* < .001 Gender (male): β = 0.172, *P* < .001 Residential status (home): β = 0.178, *P* < .001 NMSS: β = 0.176, *P* < .001
Karlstedt et al. (2017)^75^; Sweden	51	Longitudinal study database	Multiple regression	100	56.9	70.7	Caregiver: MS: β = −0.559, *P* < .001
Klietz et al. (2020)^90^; Germany	118	Community	Multiple regression	93.2	66.1	65.4	Caregiver: SF-36: β = −0.253, *P* = .013 PwP: MDS-UPDRS II: β = 0.265, *P* = .046
Klietz et al. (2020)^78^; Germany	119	Community; medical setting	Multiple regression	NR	65.5	65.4	PwP: MDS-UPDRS II: β = 0.415, *P* = .002
Klietz et al. (2020)^77^; Germany	78	NR	Multiple regression	NR	53.8	64.8	PwP: PDQ-8: β = 0.294, *P* = .064 MDS-UPDRS III: β = 0.272, *P* = .086
Kudlicka et al. (2014)^76^; UK	50	Medical setting	Backward stepwise multiple regression	69.2	NR	NR	PwP: BRIEF-A (caregiver version): β = 0.754, *P* = .000 HY: β = 0.377, *P* = .008
Leroi et al. (2012)^91^; UK	71	NR	Forced entry multiple regression	54	39.4	62.7	Apathy PwP Group: MMSE-Serial Sevens (PwP): *B* = −5.100, SE *B* = 1.335, *P* = .001 Impulse Control Disorders PwP Group: LEDD (PwP): *B* = 0.013, SE *B* = 0.005, *P* = .012 HADS-Depression (PwP): *B* = 1.677, SE *B* = 0.763, *P* = .042
Lo Monaco et al. (2021)^92^; Italy	51	Medical setting	Backward stepwise multiple regression	89	67	69	PwP: ADLs: *B* = −6.48, SE *B* = 2.84, *P* = .027
Macchi et al. (2020)^68^; United States and Canada	175	Medical setting	Stepwise multiple regression	81.7	73.1	66.1	Caregiver: HADS-Anxiety: *R*^2^ = 0.077, *P* = .0002 HADS-Depression: *R*^2^ = 0.062, *P* = .0014 PwP: FACIT-SP (Faith): *R*^2^ = 0.024, *P* = .038 PDQ-39: *R*^2^ = 0.161, *P* < .0001 QoL-AD Caregiver Reported: *R*^2^ = 0.088, *P* < .0001
Martinez-Martin et al. (2015)^93^; Spain	584	Validation study database	Stepwise multiple regression	61.2	70.5	59.6	PwP: SEND-PD (Mood/Apathy): β = 0.284, *P* < .001 SEND-PD (Psychosis): β = 0.184, *P* < .001 CISI-PD: β = 0.213, *P* < .001 Disease duration: β = 0.112, *P* = .003
Oguh et al. (2013)^74^; United States	2476	National register	Stepwise multiple logistic regression	91	NR	NR	PwP: PDQ-39 ≥47: OR = 5.1 (95% CI = 3.2, 8.2) HY: OR = 2.0 (95% CI = 1.3, 3.1) Concomitant medications: antidepressants OR = 2.1 (95% CI = 1.5, 3.1); antipsychotics OR = 2.5 (95% CI = 1.5, 4.2) Social worker visits: OR = 1.6 (95% CI = 1.2, 2.1) Gender (male): OR = 2.3 (95% CI = 1.5, 3.5) Verbal fluency: OR = 0.95 (95% CI = 0.92, 0.98)
Oh et al. (2015)^94^; South Korea	48	Medical setting	Multiple regression	NR	NR	NR	PwP: NPI-12: *B* = 0.619, SE = 0.151, *P* < .01 (ZBI) NPI-12: *B* = 0.758, SE = 0.144, *P* < .01 (CBI) Disease duration: *B* = 1.449, SE = 0.671, *P* < .05 (CBI)
Peters et al. (2011)^95^; UK	704	Community	Multiple regression	88.9	71.9	67.1	PwP: PDQ-39 (Mobility): β = 0.21, *P* < .001 PDQ-39 (Social Support): β = 0.15, *P* = .001
Rajiah et al. (2017)^96^; Malaysia	130	Community	Stepwise multiple regression	NR	69.8	45.1	PwP: PDQ-39 (Stigma and Emotional Wellbeing): *R*^2^ = 0.486, *P* = .001
Rodríguez-Violante et al. (2015)^97^; Mexico	201	Medical setting	Multiple regression	53.2	73.1	51.6	PwP: MDS-UPDRS II: β = 0.54, *P* < .007
Santos-Garcia and de la Fuente-Fernandez (2015)^98^; Spain	121	Medical setting	Multiple regression	66.9	71.9	60.2	PwP: BDI: β = 0.321, *P* = .003 SE-ADLs: β = −0.536, *P* < .0001
Sanyal et al. (2015)^99^; India	150	Medical setting	Stepwise multiple regression	48	79	50.4	Caregiver: Caregiving duration: β = 0.441, *P* = .037 PwP: HY: β = 3.493, *P* = 0.001 UPDRS III: β = 0.898, *P* < .0005
Sarandol et al. (2009)^100^; Turkey	57	Medical setting	Stepwise multiple regression	61.4	59.6	Spouse = 61.3; adult child = 43.7	PwP: GDS: *R*^2^ = 0.157, *P* < .05 BEHAVE-AD-FW: *R*^2^ = 0.145, *P* < .05
Schmotz et al. (2017)^101^; Germany	20	Community; medical setting	Multiple regression	NR	70	67.1	No significant predictors
Shin et al. (2012)^102^; South Korea	91 (spouse = 50; offspring = 41)	Medical setting	Multiple regression	54.9	Spouse = 50; offspring = 53.7	Spouse = 66.4; offspring = 45.8	Spousal Group: CES-D (caregiver): β = 0.754, *P* < .001 UPDRS I (PwP): β = 0.263, *P* = .002 Offspring Group: Community relationships (caregiver): β = −0.318, *P* = .034 UPDRS III (PwP): β = 0.455, *P* = .012
Shin et al. (2012)^103^; South Korea	42	Medical setting	Multiple regression	NR	61.9	60	Caregiver: CES-D: *B* = 1.1, SE = 0.2, *P* < .001 PwP: UPDRS I: *B* = 2.9, SE = 1.3, *P* = .03
Tanji et al. (2013)^69^; Japan and United States	178 (Japan = 83; United States = 96)	Medical setting	Multiple regression	100	NR	Japan = 68.9; United States = 63.7	Japanese Group: BDI (caregiver): β = 0.35, *P* = .003 Falls (PwP): β = 0.36, *P* = .003 United States Group: Help from others (caregiver): β = −0.34, *P* = .001 BSI (caregiver): β = 0.25, *P* = .03 BSI (PwP): β = 0.41, *P* = .001
Viwattanakulvanid et al. (2014)^104^; Thailand	85	Medical setting	Stepwise multiple regression	49.4	78.8	50.8	PwP: HADS-Anxiety: β = 0.494, *P* = .000 NADCS-Akinesia: β = 0.237, *P* = .008 MPDSS-item 14: β = −0.229, *P* = .009
Wallhagen & Brod (1997)^72^; United States	45	Medical setting; community	Multiple regression	100	69	69	PwP: Perceived control over symptoms: β = −0.29, *P* = .03
Wandrekar et al. (2014)^105^; India	50	Community	Multiple regression	76	76	NR	PwP: MDS-UPDRS I: β = 0.31, *P* < .05 MDS-UPDRS II: β = 0.23, *P* < .05 MDS-UPDRS III: β = 0.55, *P* < .05
Zhang et al. (2022)^106^; Australia	39	Medical setting; community	Multiple regression	100	61.5	67.1	Caregiver: COPE-28 (Problem-focused): β = 0.39, *P* = .02
Zhong et al. (2016)^107^; Australia	50	Medical setting	Forward stepwise multiple regression	88	68	64.7	Caregiver: HADS-Depression: *R*^2^ = 0.450, *P* < .001 PwP: NUCOG-Visuoconstructional: *R*^2^ = 0.581, *P* = .006 HADS-Depression: *R*^2^ = 0.713, *P* = .002

All studies utilized a cross-sectional design.

Abbreviations: ADL-MS: Activities for Daily Living Self-Care Scale for Multiple Sclerosis; BDI: Beck Depression Inventory; BEHAVE-AD-FW: Behavioral Pathology in Alzheimer’s Disease Frequency-Weighted Severity Scale; BRIEF-A: Behavior Rating Inventory of Executive Function—Adult Version; BSI: Brief Symptom Inventory; CBI: Caregiver Burden Inventory; CES-D: Center for Epidemiologic Studies Depression Scale; CISI-PD: Clinical Impression of Severity Index for Parkinson’s Disease; COPE-28: Brief Coping Orientation to Problems Experienced Inventory; EASI: Everyday Abilities Scale for India; FACIT-SP: Functional Assessment of Chronic Illness Therapy—Spiritual Wellbeing; GDS: Geriatric Depression Scale; GHQ-28: 28-Item General Health Questionnaire; HADS: Hospital Anxiety and Depression Scale; HY: Hoehn and Yahr Scale; LEDD: Levodopa Equivalent Daily Dose; MDS-UPDRS: Movement Disorder Society-Sponsored Revision of the Unified Parkinson’s Disease Rating Scale; MMSE: Mini Mental State Examination; MPDSS: Modified Parkinson’s Disease Sleep Scale; MS: Mutuality Scale; MSPSS: Multidimensional Scale of Perceived Social Support; NADCS: Nocturnal Akinesia Dystonia and Cramp Score; NMSS: Non-Motor Symptom Scale; NPI-12: Neuropsychiatric Inventory—12-item; NR: not reported; NUCOG: Neuropsychiatry Unit Cognitive Assessment Tool; PDQ: Parkinson’s Disease Questionnaire; PPRS: Parkinson’s Psychosis Rating Scale; PSS-Fa: Perceived Social Support from Family; QoL-AD: Quality of Life in Alzheimer’s Disease; RAS: Relationship Assessment Scale; SE-ADLs: Schwab & England Activities of Daily Living Scale; SCOPA: Scales for Outcomes in Parkinson’s Disease; SEND-PD: Scale for Evaluation of Neuropsychiatric Disorders in Parkinson’s Disease; SF-36: 36-item Short Form Health Survey; SOC: Sense of Coherence Scale; SPDDS: Self-Assessment Parkinson’s Disease Disability Score; UPDRS: Unified Parkinson’s Disease Rating Scale; ZBI: Zarit Burden Interview.

a Community recruitment included caregiver or patient support groups, newspaper adverts, and newsletters; medical settings included hospitals, outpatient clinics, palliative and primary care, and nursing homes.

Samples ranged from 20 to 2476 caregivers, with most studies (n = 35) having fewer than 200 participants. Not all studies included a definition of “informal caregiver” (n = 22). Across those that did, the definition varied. Five studies applied the definition devised by Martinez-Martin et al^108(p925)^: “any person who, without being a professional or belonging to a social support network, usually lives with the patient and, in some way, is directly implicated in the patient’s care or is directly affected by the patient’s health problem.”^74,81,93,98,104^ Other studies referred to an unpaid person who assists with daily activities or provides emotional support. Most studies did not specify a minimum time caregivers had to spend with the PwP; for those that did, requirements varied. Among studies that reported the caregiver’s relationship with the PwP (n = 34), spouses/partners dominated the sample (47%-100%), with 6 studies recruiting spouses/partners only.^69,72,75,83,84,106^ Other relationships included children, children-in-law, siblings, parents, other relatives, friends, and neighbors. Among studies reporting caregiver gender (n = 32), all but two^73,91^ of the caregiver samples were predominately female (50%-88%). Thirty-four studies reported caregivers’ mean age (43.7-70.7 years).

Seven validated measures of CB were used, including 6 non-disease-specific measures: Zarit Burden Interview (n = 25),^109^ Caregiver Burden Inventory (n = 6),^110^ Caregiver’s Burden Scale (n = 2),^111^ Caregiver Strain Index (n = 2),^112^ Feeling of Burden questionnaire (n = 1),^113^ and Multidimensional Caregiver Strain Index (n = 1),^114^ and 1 disease-specific measure, the Parkinson’s Disease Caregiver Burden Questionnaire (n = 5).^115^ All studies used regression to analyze relationships between CB (the specified dependent or outcome variable) and other variables.

### Methodological Quality Assessment

Results of the quality assessment are summarized in Table S1 (see online supplemental content). The main methodological shortcomings across included studies related to statistical analyses. While appropriate methodologies were used (ie, regression), these analyses were under-specified and/or key data (eg, type of regression, whether assumptions were fully checked and met, and which variables were included in the regression model) were missing in all but one of the studies,^76^ undermining the robustness of the approach and convincingness of findings. Fifteen studies used stepwise methods of regression, which is problematic as numerous issues have been identified with these methods (see Field^116^ and Harrell^117^ for discussion). Only one study justified the use of stepwise methods, citing the exploratory aims of the study^76^; however, given the existing available theoretical literature, it remains questionable whether stepwise regressions were the most appropriate approach to use. Furthermore, it was not uncommon across the reviewed studies for variables to be included in multivariable analysis based on their significance in bivariate analysis. This type of variable selection has been deemed inappropriate because it can result in wrongly rejecting potentially important variables that are only significant after controlling for other variables.^118^ These issues risk the development of inaccurate results.

Most studies used valid and reliable tools to measure potential predictors (n = 33) and CB (n = 39), sufficiently to limit any potential measurement bias. However, problems were identified relating to the psychometric adequacy of outcome measures used in several studies. In 3 studies, researchers modified some of the outcome measures used, but these adapted versions were not described as validated before use.^73,79,84^ Additionally, reliability and validity of outcome measures used by Martinez-Martin et al^93^ and Sanyal et al^99^ had not been clearly established for the language in which they were administered. Two studies used outcome measures originally developed for use in relation to Alzheimer’s disease^68,100^; it is unclear how these were made appropriate for use in relation to Parkinson’s. Furthermore, Tanji et al^69^ used different depression measures in their Japanese and US samples, introducing a source of potential bias. Thirty-three studies did not identify important potential confounds; however, strategies to deal with confounding variables were frequently used, given studies used a form of multiple regression analysis.

Inclusion and exclusion criteria for samples were not clearly defined in 15 studies, limiting conclusions about generalizability. Furthermore, 27 studies did not use both specified diagnostic criteria for Parkinson’s and a definition of “informal caregiver” to determine eligibility, increasing the risk of bias. Eighteen studies were appraised as not providing sufficient detail about the study sample and setting. For example, demographic information (ie, caregiver age, gender, or relationship to the PwP), important for understanding the sample and making decisions about generalizability of the results, was missing in 17 studies and 1 study did not explicitly state the setting, although this could be assumed from other information provided in the article.

### Predictors of Caregiver Burden

Evidence for predictors is reported descriptively due to the diversity of measures used across the studies. Factors predicting CB were grouped into caregiver and PwP-related factors, and thematically categorized. Due to the large number of included studies, individual study results are presented in Table 4 and key findings in relation to the most investigated predictors are described below.

**Table 4.** Variables Investigated in Relation to Caregiver Burden.

Domain	Significant predictors of higher caregiver burden	Predictors investigated but not found to be significant
Caregiver-related factors		
Demographics		• Age^47,68,69,75,81,92,93,103^ • Gender^47,75,93,103,106^ • Education^47,75,103^ • Socioeconomic status^82^ • Work status^47^ • Marital status^47^ • Living with PwP^82^
Caregiver involvement	• Greater years of caregiving^81,89^ • Greater daily hours of caregiving^99^	• Years of caregiving^68,82,107^ • Frequency of providing care^47^ • Time of day care provided^47^ • Weekly hours of caregiving^82^ • Daily hours of caregiving^107^
Psychological factors	• Greater depression^68,69,73,103,107^ - Specifically in spousal (but not offspring) caregivers^102^ • Greater anxiety^68,81^ • Psychological well-being^71^	• Anxiety^107^ • Psychological well-being^84^
Protective factors	• Low sense of coherence^73,87^ • Use of problem-focused coping strategies^47,88,106^ • Use of avoidant coping strategies^47^ • Poorer perceived social support^47,84^ - Specifically from community in offspring caregivers^102^ • Less help from others in US (but not Japanese) sample^69^ • Fewer caregivers involved in supporting PwP^79,86^ • Poorer relationship satisfaction^88^ • Poorer mutuality^75^	• Self-esteem^87^ • Social competence^87^ • Avoidant coping style^106^ • Emotion-focused coping style^47,106^ • Social support^102^ • Help from others^82^ • Relationship quality^106^ • Marital satisfaction^84^ • Spiritual well-being^68^ • Activation^47^ • Mindfulness^90^ • Psychological flexibility^90^
Other factors	• Current health problems impacting ability to provide care^88^ • Poorer sleep^88^ • Poorer health-related QoL^90^	• Medical comorbidities^69^ • Health-related QoL^102,103^
Person with Parkinson’s-related factors		
Demographics	• Older age^86^ • Male gender^70,74^ • Occupational status (not working)^47^ • Living at home (as opposed to a nursing home)^70^	• Age^73,81,82,92,94,97,103^ • Gender^73,81,90,94,97,103^ • Education^47,75,103^
Disease factors	• Advancing disease stage^73,74,76,83,86,93,99^ • Greater disease duration^86,87,93^ - Specifically using Caregiver Burden Inventory (but not Zarit Burden Inventory)^94^ • Presence of concomitant medications^74^ • Higher dopaminergic load in PwP presenting with impulsivity (but not apathy)^91^	• Disease stage^79,82,94,97,98,102,103,106,107^ • Disease duration^47,78,82,90,92,97,98,107^ • Symptom burden^68^ • Presence of atypical parkinsonism^68^ • Presence of dementia^93^ • Medication use^68,69^ • Dopaminergic load^94^
Motor symptoms	• Greater motor symptomology^47,78,90,97,105^ • Greater motor symptom severity^77,79,99,105^ - specifically in offspring (but not spousal) caregivers^102^ • Greater dependence in ADLs^47,84,89,92,98^ • Greater difficulty with eating, bathing, and meals^88^ • Higher frequency of falls in Japanese (but not US) sample^69^	• Motor symptoms^79,102,103^ • Motor symptom severity^68,75,92,97,98,101,103^ • Motor complications^79,97,98,102,103,105^ • ADL^69,79,81,92,102,103^ • Instrumental ADL^92^ • Overall functional status^68^ • Speech, gait, postural instability, freezing, fluctuation, dyskinesia, dystonia, and the presence of autonomic symptoms^69,79^
Non-motor symptoms	• Greater non-motor symptomology^70,103,105^ - Specifically in spousal (but not offspring) caregivers^102^ • Greater severity of neuropsychiatric symptoms^70,71,83,86,89,94^ - All subscales except apathy^86^ - Motor disturbance subscale only^88^ • Greater severity on psychotic symptoms subscale^93^ and mood/apathy but not impulse control disorders subscale^85,93^ • Greater depression^79,98,100,107^ - Specifically in US sample^69^ and PwP with impulse control disorders (but not apathy)^91^ • Greater anxiety^104^ • Greater impairment in visuo-constructional skills^107^ • Greater impairment in attentional ability in PwP with apathy (but not impulse control disorders)^91^ • Decreased verbal fluency^74^ • Decreased phonemic (but not semantic) verbal fluency^87^ • Presence of levodopa-induced psychotic symptoms^81^ • Greater behavioral difficulties^100^ • Greater caregiver-rated (but not PwP-rated) executive function-related behavioral problems^76^ • Greater sleep disturbances^79,81^ Presence of nocturnal akinesia and PwP feeling tired and sleepy upon awakening^104^	• Non-motor symptoms^75,97,98^ • Psychiatric symptoms^79^ • Alexithymia^78^ • Depression^47,68,76,78,81,94,101^ • Anxiety^47,68,94,107^ • Cognitive function^68,69,76,81,82,87,94,101-103,105^ • Specifically caregiver perspective on cognitive decline^75^ • Memory^87,107^ • Executive functioning^107^
Quality of life	• Poorer QoL from caregiver (but not PwP) perspective^68^ • Poorer health-related QoL^68,74,77,80,86^ • Mobility,^88,95^ social support,^95^ stigma,^96^ and emotional well-being^96^ subscales only	• Health-related QoL^78,90^
Other factors	• Less perceived control over Parkinson’s symptoms^72^ • Poorer spiritual well-being^68^ • Utilization of social or mental health visits^74^	• Perceived control over disease progression^72^ • A&E visits^68^ • Receiving home health services^68^ • Medical comorbidities^69,82,94^

Abbreviations: ADL: activities of daily living; PwP: people with Parkinson; QoL: quality of life.

### Caregiver-Related Factors

#### Demographics

Eight studies found caregiver age did not predict CB.^47,68,69,75,81,92,93,103^ Caregiver gender was consistently found unrelated to CB in 5 studies investigating this relationship.^47,75,93,103,106^ Level^47,75^ and years^103^ of caregivers’ education did not predict CB.

#### Caregiver involvement

Seven studies investigated the association between caregiver involvement and CB. Two studies found more years providing care predicted CB,^81,89^ but 3 other studies found no association.^68,82,107^ Frequency of providing care and whether care is provided only during the day, or during day and night, did not predict CB.^47^ Hours per week providing care also did not predict CB,^82^ and neither did hours per day according to Zhong et al^107^; however, Sanyal et al^99^ reported hours per day did predict CB.

#### Psychological factors

Nine studies explored the contribution of caregiver mental health to CB. Five studies found CB was positively predicted by caregiver depression.^68,69,73,103,107^ Shin et al^102^ found between-group differences regarding caregiver-PwP relationship, reporting caregiver depression predicted burden in spouses supporting a partner with Parkinson’s, but not for those supporting a parent. Two studies found caregiver anxiety positively predicted CB,^68,81^ but 1 study found no association.^107^ Johnson et al^71^ found the psychological well-being of caregivers positively predicted CB; however, Edwards and Scheetz^84^ found the psychological well-being of caregivers did not contribute to burden.

#### Protective factors

Reviewed studies explored the relevance of various protective factors including personality, coping styles, social support, and relationship quality to CB. Regarding caregiver personality, 2 studies reported higher CB was predicted by lower sense of coherence in caregivers.^73,87^ Caregivers’ self-esteem and social competence did not predict CB.^100^

Caregivers’ coping styles were explored in 3 studies. CB was consistently positively predicted by the use of problem-focused coping strategies.^47,88,106^ An avoidant coping style positively predicted CB in 1 study,^47^ but not another.^106^ Emotion-focused coping strategies did not predict CB.^47,106^

Different types of social support were explored in 7 studies. Two studies found higher perceived support from family, friends, and significant others was associated with reduced CB.^47,84^ Shin et al^102^ assessed social support regarding the degree of assistance caregivers received from private relationships (eg, family, friends, neighbors) and the community (eg, organizations or experts). Community support negatively predicted CB in caregivers of a parent with Parkinson’s, but private support did not predict burden for caregivers of either a spouse or a parent with Parkinson’s. Carrilho et al^82^ found whether the caregiver received help from others did not predict CB. Tanji et al^69^ found receiving help negatively predicted burden in the main caregiver in a group of US-based caregivers, but not for caregivers living in Japan. In 2 other studies, CB in the main caregiver was negatively predicted by number of caregivers involved in supporting the PwP.^79,86^

Factors linked to the caregiver-PwP relationship were investigated in 4 studies. General relationship satisfaction^88^ and mutuality^75^ negatively predicted CB. However, no association was found between CB and relationship quality^106^ and marital satisfaction.^84^

### Person With Parkinson’s-Related Factors

#### Demographics

Eight studies examined PwP age; most found this did not predict CB; ^73,81,82,92,94,97,103^ however, 1 study reported a positive predictive effect of PwP age.^86^ Six studies found PwP gender did not predict CB,^73,81,90,94,97,103^ but 2 studies reported male gender was associated with higher CB.^70,74^ Level^47,75^ and years^103^ of the PwP education did not predict CB.

#### Disease factors

The relationship between disease stage and CB was explored by multiple studies, with mixed findings. While 7 studies found advancing disease stage positively predicted CB,^73,74,76,83,86,93,99^ 9 studies found no association.^79,82,94,97,98,102,103,106,107^

Twelve studies explored the contribution of disease duration to CB. Eight studies did not identify disease duration as a significant predictor.^47,78,82,90,92,97,98,107^ However, 3 studies found disease duration positively predicted CB.^86,87,93^ Oh et al^94^ found disease duration was a predictor when burden was measured using the Caregiver Burden Inventory but not the Zarit Burden Interview.

#### Motor symptoms

The relationship between CB and motor symptoms as assessed by the original Unified Parkinson’s Disease Rating Scale (UPDRS) and the Movement Disorder Society-Sponsored Revision of the Scale (MDS-UPDRS) was extensively investigated in the studies. Eight studies utilized the UPDRS II or MDS-UPDRS II to assess motor symptoms. UPDRS II scores did not predict CB,^79,102,103^ whereas higher scores on the MDS-UPDRS II did contribute to greater CB.^47,78,90,97,105^

Twelve studies utilized the UPDRS III and MDS-UPDRS III to assess motor symptom severity. Among studies using the former, 6 found motor symptom severity did not predict CB,^68,75,92,98,101,103^ 2 found it did,^79,99^ and Shin et al^102^ concluded motor symptom severity positively predicted CB in offspring but not spousal caregivers. Regarding the MDS-UPDRS III, 2 studies found scores positively predicted CB^77,105^ and 1 did not identify a relationship.^97^

The UPDRS IV and the MDS-UPDRS IV were used by 4 and 2 studies, respectively, assessing associations between motor complications and CB. No studies found these predicted CB.^79,97,98,102,103,105^

#### Functional status

Numerous studies reported on the effect of functional ability (assessed by various ADL scales) on CB. Findings were variable. Four studies reported greater dependence in ADLs predicted higher CB^47,84,89,98^; 5 did not identify an association.^69,79,81,102,103^ Lo Monaco et al^92^ found caregiver reports regarding the extent to which they provide help in ADLs, but not score on an ADL scale, predicted burden. CB was not predicted by instrumental ADLs^92^ and overall functional status.^68^ Hand et al^88^ found greater difficulty specifically with eating, bathing, and mealtime tasks predicted CB.

#### Non-motor symptoms

Seven studies used various overall measures of non-motor symptoms to investigate their relationship to CB. Three studies reported CB was positively predicted by non-motor symptoms,^70,103,105^ one concluded non-motor symptoms predicted burden in caregivers of a spouse but not a parent with Parkinson’s,^102^ and 3 found no association.^75,97,98^

Eleven studies explored associations between neuropsychiatric symptoms and CB. Six studies found more severe neuropsychiatric symptoms overall predicted higher CB,^70,71,83,86,89,94^ while 1 study found no association.^79^ Furthermore, Goel et al^86^ demonstrated specific neuropsychiatric symptoms (delusions, hallucinations, agitation, depression, anxiety, elation, disinhibition, irritability, motor disturbance, nighttime behaviors, and appetite/eating) except apathy independently predicted CB. Conversely, Hand et al^88^ found motor disturbance was the only domain to positively predict CB. Two studies found greater severity of mood/apathy symptoms (but not impulse control disorders) predicted greater CB.^85,93^ Martinez-Martin et al^93^ additionally found psychotic symptoms positively predicted CB.

#### Depression

Thirteen studies explored relationships between PwP depression and CB, and evidence was mixed. Four studies found PwP depression positively predicted CB.^79,98,100,107^ Tanji et al^69^ concluded feelings of depression was a predictor in a US sample of caregivers. Leroi et al^91^ found more severe PwP depression predicted higher CB only in individuals with Parkinson’s with impulse control disorders, and not in those with apathy. Seven studies did not identify PwP depression as a predictor.^47,68,76,78,81,94,101^

#### Anxiety

PwP anxiety was investigated in only 5 studies. One found higher PwP anxiety predicted greater CB,^104^ while 4 found no association.^47,68,94,107^

#### Cognition

The impact of cognitive impairment on CB was extensively explored. Global cognitive function was assessed in 11 studies, all finding this did not predict CB.^68,69,76,81,82,87,94,101-103,105^ Furthermore, Karlstedt et al^75^ reported caregiver perspectives on cognitive decline in the PwP did not predict burden.

Specific cognitive domains including memory (short-term and working memory)^87,107^ and executive functioning^107^ were also not found to predict CB. However, greater impairment in visuo-constructional skills was predictive of burden.^107^ Leroi et al^91^ found attentional ability negatively predicted CB in PwP with apathy, but not with impulse control disorders. Oguh et al^74^ found decreased verbal fluency predicted higher CB, while Golińska et al^87^ reported better phonemic (but not semantic) verbal fluency predicted less CB.

#### Sleep

PwP sleep problems were sparsely explored. Two studies identified sleep disturbances positively predicted CB.^79,81^ Furthermore, Viwattanakulvanid et al^104^ found nocturnal akinesia and PwP feeling tired and sleepy upon awakening predicted higher CB.

#### Quality of life

Macchi et al^68^ found poorer QoL from the caregiver’s perspective, but not the PwP, contributed to higher CB. Self-reported health-related QoL positively predicted CB in 5 studies,^68,74,77,80,86^ although this association was not found by Klietz et al^90^ and Klietz et al.^78^ In studies investigating specific aspects of health-related QoL, CB was positively predicted by only mobility,^88,95^ social support,^95^ stigma,^96^ and emotional well-being.^96^

## Discussion

This review provides an updated appraisal and synthesis of quantitative research investigating caregiver and PwP-related predictors of CB in informal caregivers of PwP. Certain variables consistently predicted increased burden including greater impact of motor symptoms on daily living, greater severity of neuropsychiatric symptoms, poorer QoL of the PwP, and poorer caregiver mental health. Evidence was inconclusive for many variables including disease stage and duration, motor symptom severity, functional ability, non-motor symptoms, mental health of the person with Parkinson’s, and caregiver involvement and protective factors. However, demographics, presence of motor symptoms, motor complications, and general cognitive function were consistently not found to be predictors. Crucial points were also identified to improve robustness and consistency across the literature going forward, which may help define the contribution of inconclusive variables. Across the identified studies, many failed to define “informal caregiver” or applied varying definitions, 7 different CB measures were employed, and outcome measures examining predictors of experienced burden varied widely.

Disease stage, disease duration, motor symptom severity, motor complications, and functional ability were among the most-explored variables. Of these, only motor complications were consistently non-predictive. Findings were mixed for the other variables. This contradicts Greenwell et al’s^54^ review, which concluded disease stage and disease duration rarely predicted burden—although this review is now significantly outdated, and new evidence included here may have led to different conclusions. The less consistent findings in the current review may suggest the impact of these factors on burden is mediated or moderated by other variables, and that variables cannot effectively be considered in isolation.^119^ While impossible to tell from the information available in this review, it does indicate the importance of using theoretical models to inform study design and data analysis, and multivariable analysis, as this allows multiple factors to be taken into account and to consider confounds between variables.

Influences of motor symptoms on CB were also commonly explored. Outcomes of studies utilizing the UPDRS II versus MDS-UPDRS II to explore their influences were strikingly different. Studies utilizing the former found motor symptoms non-predictive of burden; studies using the latter identified it as predictive. This disparity may be explained by differences in the measures themselves. While there is general parallelism between the original UPDRS II and the MDS-UPDRS II, the original focuses on *presence* of motor symptoms whereas the MDS-UPDRS II focuses on their *impact* on ADLs.^120^ It appears, therefore, only the latter influences burden, which stresses the importance of providing support for caregivers caring for PwP whose motor symptoms significantly impact their daily living. Interestingly, the same difference applies to the UPDRS I and MDS-UPDRS I used for assessing non-motor symptoms (the MDS-UPDRS I focuses on impact rather than presence of non-motor symptoms), although findings varied more across studies using these measures.

Most evidence indicated neuropsychiatric symptoms predicted higher burden. The only study that found no association did not use a disease-specific measure,^79^ potentially explaining their differential finding. This contradicts Greenwell et al’s^54^ review findings of limited, mixed evidence for this predictor. Again, the growth in literature has perhaps provided clarity about relationships between these variables. Regarding specific neuropsychiatric symptoms, evidence consistently implies global cognitive function does not predict burden, but specific cognitive deficits may contribute to burden. Further research is required to draw a more certain conclusion, given the current limited evidence. Overall, these findings indicate CB could be prevented or alleviated by adequate management of neuropsychiatric symptoms. The contribution of PwP QoL to burden was little-explored by Greenwell et al^54^; however, the current literature suggests poorer PwP QoL predicts higher CB.

Studies exploring contributions of caregiver involvement to burden achieved inconsistent results. Although the various ways this variable was conceptualized and measured could somewhat explain the mixed findings, there were also disparities across studies using the same measures. An alternative explanation for these inconsistencies is that effects of caregiver involvement on burden appear mediated by other variables, such as frequency of breaks and perceived social support.^119^ Furthermore, caregiver involvement was measured subjectively, based on caregivers’ interpretations of which activities and roles constitute caregiving^54^; this reflects wider disagreements over what formally constitutes “caregiving” as opposed to non-bureaucratized support of family.^26,27^ Cultural considerations add to this uncertainty, as many activities considered formalized caregiving in one culture might be regarded as typical family support in another.^20,21^

Multiple studies found caregivers’ psychological difficulties predicted increased burden. However, conceptual confounding is likely since emotional problems are an important aspect of burden^19^ and relevant questions are often included in CB measures (eg, Caregiver Burden Inventory—“I feel emotionally drained due to caring for him/her”).^110^ Although in reviewed studies caregiver depression and anxiety were entered as predictors in statistical models, it is difficult to disambiguate the relationship between these variables. Nevertheless, results emphasize the importance of ensuring psychological support is available to caregivers.

Caregiver social support was frequently investigated and predicted burden in most studies. However, studies were again inconsistent regarding conceptualizations and measurement of social support, meaning findings should be interpreted cautiously. As for defining “caregiving,” social support is multifaceted and includes varied formal and informal support. The type of social support found helpful differed according to the caregiver’s relationship with the PwP, and the cultural background of the caregiver and PwP influenced whether social support was a predictor. Further research is required to identify the most effective ways to develop and maintain support (perhaps by subgroup). One systematic review^54^ suggested perceptions, not actual support, is influential, but this was not confirmed here.

This review found factors contributing to perceived burden could vary between subsamples (eg, people supporting a spouse versus parent, different cultural backgrounds, certain Parkinson’s presentations, and caregiver versus PwP perspective on the variable). Although the evidence base is limited regarding subsample analyses, these findings have important implications for tailoring approaches to reduce CB for people from different subgroups.

### Limitations

There were methodological weaknesses in the studies that underpin this review. Caregiving is a dynamic process, but the cross-sectional included studies provide a more static picture and consequently limited insight into the trajectory of CB and its predictors over time.^121^ Cross-sectional designs also inhibit conclusions regarding causal relations.^122^ Furthermore, although multivariable analysis assumes cause-and-effect relationships between variables, some associations explored may be multifaceted. Included studies rarely developed predictive models referencing theoretical literature; instead, most variables investigated appeared to be chosen relatively arbitrarily or at least were not explicitly justified. While the use of validated outcome measures is considered a strength of the reviewed studies, it is also important to acknowledge these measures may be biased toward the research perspective and consequently may not capture all aspects of the patient or caregiver experience.

Regarding power, multivariable analysis requires large samples; however, many studies reported relatively small (and potentially underpowered—only 3 studies reported power calculations^75,96,106^) samples, reducing their ability to detect relationships. Definitions of “informal caregiver” varied or were lacking, so there was likely between-study variation regarding type and level of care provided. Predictors of burden may differ depending on the caregiving role, but due to ambiguity in the study set regarding caregiver sample definitions, this cannot be determined for the current review. Samples also predominantly comprised older females caring for a spouse/partner, although this may reflect the older age of disease onset, that Parkinson’s disproportionately affects males, and that spouses/partners are common sources of support.

While it is acknowledged a limitation of the present review is that data on disease severity or stage were not extracted from included studies, generalization of findings to the wider Parkinson’s population is questionable because most studies recruited from neurology outpatient clinics or similar settings. Samples were therefore likely biased toward caregivers supporting individuals earlier in the course of Parkinson’s, as later motor impairments present challenges for attending appointments or continuing to live in the community rather than institutionalized care.^123^ Experiences of the identified samples may further differ from those in remote areas with restricted access to health care, or from those who choose not to access it. Any impact of Parkinson’s severity on CB would consequently be difficult to evaluate across populations less well-represented given this recruitment bias, yet research has shown those supporting people in the palliative stage of Parkinson’s may experience particular physical and emotional stress.^12^ Although 21 countries were represented in this review, the vast majority were countries with predominantly WEIRD (Western, Educated, Industrial, Rich, Democratic) populations, and so the evidence base provides a biased understanding of CB among those supporting PwP.

Limitations of this systematic review include that the literature search was restricted to articles published in English, and so the findings are subject to a language and cultural bias. Also, only full-text, peer-reviewed published studies were included, and therefore, evidence from gray literature might have been missed. Finally, it is acknowledged only 1 author assessed the methodological quality of included studies, which may have affected the reliability of the quality appraisal.

### Future Directions

Future research should investigate caregiver-related variables including personality, coping style, and caregiver-PwP relationship, as these are understudied and are potentially modifiable through intervention. It will also be useful to explore perceived positive aspects of caregiving as these are sparsely investigated but may also be important predictors of CB and contribute to developing interventions to support caregivers. New research should specify their definition of “informal caregiver” and ideally employ a more consistent definition across studies. Future research needs to increase inclusion from younger caregivers and other relationship categorizations (eg, parent-child, or between siblings), as well as recruit a more diverse sample regarding disease severity, to provide a more comprehensive understanding of experiences of all informal caregivers of PwP. Conducting subsample analyses would help to better understand whether factors predicting CB differ depending on demographic characteristics of caregivers, which could also facilitate the development of an evidence base to inform tailored interventions for people from different subgroups. Furthermore, future studies would benefit from using objective and validated measures of caregiver involvement. They might also consider using disease-specific outcome measures to assess aspects of burden relevant to the specific population and develop an understanding of needs in this population for future interventions. Finally, new studies should ideally use longitudinal designs to explore the dynamic nature of CB in Parkinson’s and its predictors, to evaluate causation between identified variables and burden—though this may be constrained by financial and practical difficulties.

## Conclusion

This systematic review provides an up-to-date synthesis and critical evaluation of the evidence base around predictors of CB among caregivers of PwP. There were reasonably clear and well-supported findings for certain risk factors for CB that can inform future care and support, specifically caregiver mental health, impact of motor symptoms on ADLs, neuropsychiatric symptoms, and PwP QoL. Key methodological weaknesses across the evidence base are also identified with recommendations for addressing them, offering routes to improved robustness and consistency in future relevant research. Findings from this review can contribute to the development of targeted interventions to prevent or alleviate possible negative impacts of caregiving and potentially help identify caregivers particularly at risk of higher CB that could be prioritized in the context of under-resourced services.

## Supplemental Material

sj-pdf-1-wjn-10.1177_01939459251327968 – Supplemental material for Predictors of Informal Caregiver Burden in Parkinson’s Disease: A Systematic Review Supplemental material, sj-pdf-1-wjn-10.1177_01939459251327968 for Predictors of Informal Caregiver Burden in Parkinson’s Disease: A Systematic Review by Rosie Lesley, Jane Simpson, Maria Dale, Fiona Eccles, Selina Lock and Sarah Gunn in Western Journal of Nursing Research
