# Supplementary material for: Predictors of Informal Caregiver Burden in Parkinson’s Disease: A Systematic Review
Source: West J Nurs Res. 2025 Mar 26;47(6):524–43. doi: 10.1177/01939459251327968 (PMC12069830; doi:10.1177/01939459251327968)
Supplement: sj-pdf-1-wjn-10.1177_01939459251327968 – Supplemental material for Predictors of Informal Caregiver Burden in Parkinson’s Disease: A Systematic Review [file sj-pdf-1-wjn-10.1177_01939459251327968.pdf]

Table S1. Quality Appraisal of Included Studies Using the JBI Critical Appraisal Checklist for Analytical Cross-Sectional Studies

	Criteria for inclusion in the sample clearly defined	Study subjects and the setting described in detail	Exposure measured in a valid and reliable way	Objective, standard criteria used for measuring the condition	Confounding factors identified	Strategies to deal with confounding factors stated	Outcomes measured in a valid and reliable way	Appropriate statistical analysis used
Agrawal et al. (2012)[95]	Unclear	Yes	Unclear	Yes	No	Unclear	Yes	Unclear
Bartolomei et al. (2018)[96]	Yes	Unclear	Yes	No	No	Unclear	Yes	Unclear
Caap-Ahlgren and Dehlin (2002)[73]	Unclear	Yes	Unclear	No	No	Yes	Yes	Unclear
Carod-Artal et al. (2013)[81]	Yes	Yes	Yes	Yes	No	Yes	Yes	Unclear
Carrilho et al. (2018)[97]	Yes	Yes	Yes	No	No	Yes	Yes	Unclear
D'Amelio et al. (2009)[86]	No	No	Yes	No	No	Yes	Yes	Unclear
Edwards and Scheetz (2002)[85]	No	Yes	Unclear	No	No	Yes	Yes	Unclear
Eichel et al. (2022)[98]	Yes	Yes	Yes	No	Yes	Yes	Yes	Unclear
Geerlings et al. (2023)[47]	Yes	Yes	Yes	Yes	No	Yes	Yes	Unclear
Goel et al. (2022)[99]	Yes	No	Yes	Yes	No	Yes	Yes	Unclear
Golińska et al. (2017)[100]	Yes	No	Yes	No	No	Yes	Yes	Unclear
Hand et al. (2022)[101]	Yes	Yes	Yes	No	No	Yes	Yes	Unclear
Johnson et al. (2023)[71]	Yes	No	Yes	No	No	Yes	Yes	Unclear
Jose et al. (2021)[102]	No	No	Yes	No	No	Yes	Yes	Unclear
Kalampokini et al. (2022)[70]	Yes	No	Yes	No	No	Yes	Yes	Unclear
Karlstedt et al. (2017)[75]	Yes	Yes	Yes	No	Yes	Yes	Yes	Unclear
Klietz et al. (2020)[103]	Yes	Yes	Yes	No	No	Yes	Yes	Unclear
Klietz et al. (2020)[78]	Yes	No	Yes	No	Unclear	Yes	Yes	Unclear
Klietz et al. (2020)[77]	Yes	No	Yes	No	No	Yes	Yes	Unclear
Kudlicka et al. (2014)[76]	Yes	No	Yes	No	No	Yes	Yes	Yes

Leroi et al. (2012)[87]	Yes	No	Yes	Unclear	No	Yes	Yes	Unclear
Lo Monaco et al. (2021)[104]	Yes	Yes	Yes	No	No	Yes	Yes	Unclear
Macchi et al. (2020)[68]	Yes	Yes	Unclear	No	No	Yes	Yes	Unclear
Martinez-Martin et al. (2015)[80]	Yes	Yes	Unclear	Yes	No	Yes	Unclear	Unclear
Oguh et al. (2013)[74]	Unclear	No	Yes	Yes	Yes	Yes	Yes	Unclear
Oh et al. (2015)[105]	Unclear	No	Yes	Unclear	No	Yes	Yes	Unclear
Peters et al. (2011)[106]	No	Yes	Yes	No	Yes	Yes	Yes	Unclear
Rajiah et al. (2017)[107]	Yes	No	Yes	No	No	Yes	Yes	Unclear
Rodríguez-Violante et al. (2015)[108]	No	Yes	Yes	Yes	No	Yes	Yes	Unclear
Santos-García and De La Fuente-Fernández (2015)[83]	Yes	Yes	Yes	Yes	Yes	Yes	Yes	Unclear
Sanyal et al. (2015)[109]	No	Yes	Unclear	No	No	Yes	Unclear	Unclear
Sarandol et al. (2009)[110]	No	Yes	Unclear	No	No	Yes	Yes	Unclear
Schmotz et al. (2017)[111]	Yes	No	Yes	Unclear	No	Yes	Yes	Unclear
Shin et al. (2012)[112]	No	Yes	Yes	No	No	Yes	Yes	Unclear
Shin et al. (2012)[113]	No	No	Yes	Yes	Yes	Yes	Yes	Unclear
Tanji et al. (2013)[69]	Yes	No	No	No	No	Yes	Yes	Unclear
Viwattanakulvanid et al. (2014)[82]	Yes	Yes	Yes	Yes	No	Yes	Yes	Unclear
Wallhagen and Brod (1997)[72]	Yes	Yes	Yes	No	Yes	Yes	Yes	Unclear
Wandrekar et al. (2014)[114]	Unclear	No	Yes	Unclear	No	Yes	Yes	Unclear
Zhang et al. (2022)[84]	Yes	Yes	Yes	No	No	Yes	Yes	Unclear
Zhong et al. (2016)[115]	No	Yes	Yes	No	No	Yes	Yes	Unclear
